# BCL-2 (-938C>A), BAX (-248G>A), and HER2 Ile655Val Polymorphisms and Breast Cancer Risk in Indian Population

**DOI:** 10.1155/2021/8865624

**Published:** 2021-02-25

**Authors:** Deepti Bhatt, Amit Kumar Verma, Prahalad Singh Bharti, Yamini Goyal, Mohammed A. Alsahli, Ahmad Almatroudi, Arshad Husain Rahmani, Saleh Almatroodi, Prakash C. Joshi, Mohammad Mahtab Alam, Irfan Ahmad, Gaffar Sarwar Zaman, Kapil Dev

**Affiliations:** ^1^Department of Biotechnology, Jamia Millia Islamia, New Delhi, India; ^2^Department of Biophysics, All India Institute of Medical Sciences, New Delhi, India; ^3^Department of Medical Laboratories, College of Applied Medical Sciences, Qassim University, Buraidah, Saudi Arabia; ^4^Department of Zoology and Environmental Sciences, GKV, Haridwar, India; ^5^Department of Basic Medical Sciences, College of Applied Medical Sciences, King Khalid University, Abha, Saudi Arabia; ^6^Department of Clinical Laboratory Sciences, College of Applied Medical Sciences, King Khalid University, Abha, Saudi Arabia

## Abstract

Breast cancer is the most common carcinoma in women worldwide. The present case-control study was aimed to examine the association of BCL-2 (-938C> A), BAX (-248G > A), and HER2 (I655V i.e. A > G) polymorphisms with breast cancer risk in Indian population. This study enrolled 117 breast cancer cases and 104 controls. BCL-2 (-938C > A), BAX (-248G > A), and HER2 Ile655Val polymorphisms were screened by PCR-RFLP method. There was no significance difference in the allelic and genotype frequency of the BCL-2 (-938C > A) and BAX (-248G > A) polymorphisms between cases and controls. In relation to HER2 Ile655Val polymorphism, the statistical analysis of observed genotypic frequencies showed significant association (*p*-0.0059). Compared to Ile/Ile (A/A) genotype, frequency of Ile/Val (A/G) genotype was significantly higher among cases than in control group and observed to increase the breast cancer risk (OR, 2.43; 95%CI, 1.32–4.46; *p*-0.004). The frequency of Val (G) allele was significantly higher in cases as compared to controls (6.83% vs 2.88%, resp.). Compared to Ile (A) allele, significant increase in the risk of breast cancer was observed with Val (G) allele (OR, 2.21; 95% CI, 1.35–3.63; *p*-0.0016). We observed significant association between HER2 Ile655Val polymorphism and breast cancer risk under the dominant (OR = 2.52; 95% CI: 1.41–4.51; *p*-0.001) and codominant (OR, 2.24; 95% CI: 1.23–4.09; p-0.008) model. In our study, BCL-2 (-938C > A) and BAX (-248G > A) polymorphism were not found to be associated with breast cancer risk. This present study for the first time shows significant association of HER2 Ile655Val polymorphism with risk of breast cancer in Indian population. Therefore, we suggest that each population need to evaluate its own genetic profile for breast cancer risk that may be helpful for better understanding the racial and geographic differences reported for breast cancer incidence and mortality.

## 1. Introduction

Breast cancer is the leading cause of cancer-related deaths and it is the most common type of cancer among women worldwide [1]. In India, projected number of breast cancer cases is 179,790 in the year 2020 and will comprise approximately 10% of all cancers [2]. Various risk factors are associated with the development, pathogenesis, and progression of breast cancer, including genetic, environmental, biological, and lifestyle factors [3]. The relation between the occurrence of a cancer and the existence of genetic alterations is now well established [4]. For better understanding the etiology of breast cancer, recent approaches involve the molecular markers identification, which may help in prediction and prognosis of the disease [5, 6]. Apoptosis and cellular proliferation have a significant role in normal development and carcinogenesis of mammary gland [7]. Delicate homeostasis between apoptosis and proliferation in normal tissues is maintained by variety of proteins of the BCL-2 family. The BCL-2 family of proteins is divided into two main classes, proapoptotic members like BAX (BCL-2-associated X protein) and BAK, and antiapoptotic members like BCL-2 (B-cell leukemia/lymphoma 2) and BCL-xL [8]. BCL-2 gene is located on chromosome 18q21.3 [9] and comprises of three exons and two promoters (P1 and P2), both having different functions. The BAX gene is mapped to chromosome 19q13.3 q13.4 [10]. Dysregulation in the BCL-2 and BAX genes expression may cause disruption of cellular homeostasis and origin of malignancy. The functional promoter polymorphisms in BCL-2 and BAX genes were found to change the protein expression or function that may have an effect on the delicate balance in mechanisms which regulate apoptosis.

Human epidermal growth factor receptor 2 (HER2/neu/EGFR2/ERBB2/c-erbB-2) protooncogene encodes a 185 kDa transmembrane glycoprotein [11, 12] which plays important role in cell growth regulation, differentiation, and survival [13]. To date, no study has ever been conducted to evaluate the association of HER2 polymorphism with breast cancer risk in Indian population. Although the role of BCL-2, BAX, and HER2/neu is established in breast cancer pathogenesis, the exact molecular mechanism is still not clear. Therefore, the aim of the present case-control study was to investigate the association of BCL-2 (-938C>A), BAX (-248G>A), and HER2 Ile655Val polymorphisms with breast carcinoma risk in Indian population.

## 2. Materials and Methods

### 2.1. Study Subjects

In the current case-control retrospective study, a total of 117 cases of primary breast cancer were included, which fulfilled the relevant selection criteria, and a total 104 nonmalignant lesions cases of the breast tissue were taken as control after obtaining the ethical clearance from Institute Ethics Committee (Proposal no. 27/07/2017/GKV/IEC/2017). Inclusion criteria were that the required tissue sample was retrieved from the paraffin blocks prepared from primary breast tumor site only cases, which were diagnosed as infiltrating ductal carcinoma, not otherwise specified (IDC, NOS). Exclusion criteria were patients with history of recurrence of breast tumor (only cases of primary breast carcinoma were included in the study), history of prior radiation exposure to the site (prior radiotherapy) and history of neoadjuvant chemotherapy. The sample size was estimated by using the following formula: *N* = *Z*^2^*P*(1 − *P*)/*d*^2^ (where *N* is sample size, *P* is expected prevalence, *Z* is the statistic corresponding to level of confidence, and *d* is precision (corresponding to effect size)). The written informed consent was collected from all participating subjects/individuals. The relevant clinical history of all the cases of the study was collected and clinical history was used for the selection of appropriate cases as per exclusion/inclusion criteria of the study. The mean age of cases was 48.69 years and median age was 48 years. Cases had age range between 18 and 73 years and age group of 45 to 60 years had a peak prevalence rate.

### 2.2. DNA Isolation and Genotyping

Genomic DNA was isolated from paraffin embedded tumor tissue blocks by phenol-chloroform method. Genotyping of the SNPs BCL-2-938C>A, BAX-248G>A, and HER2 (I655V, i.e., A>G) was performed by using the polymerase chain reaction-restriction fragment length polymorphism (PCR-RFLP) assay. PCR reactions were performed in a 25 *µ*l reaction mixture containing 1 *µ*l genomic DNA, 10X PCR buffer 2.5 *µ*l, 2.5 *µ*l dNTP, 0.5 *µ*l of each primer, and 1 *µ*l Taq DNA polymerase. For BCL-2, PCR conditions include initial denaturation at 96°C for 5 min followed by 35 cycles at 96°C–for 45 seconds, at 56°C for 45 seconds, and at 72°C for 30 s and a final extension step at 72°C for 10 minutes. For BAX, PCR conditions include initial denaturation at 95°C for 5 min followed by 35 cycles at 95°C–48 seconds, at 54°C for 45 seconds, and at 72°C for 40 s and a final extension step at 72°C for 8 minutes. For HER2, PCR conditions include initial denaturation at 94°C for 5 min followed by 35 cycles at 94°C–30 s, at 62°C–45 seconds, and at 72°C for 30 s and a final extension step at 72°C for 7 minutes.

**Figure 1:**
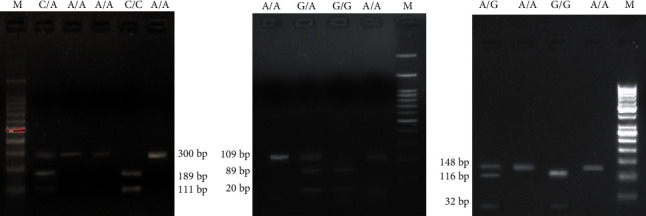
2% agarose gel electrophoresis for digested PCR product. M-Ladder (a) BCL-2, Hetero (C/A), Homo mutant (A/A), and Homo wild (C/C); (b) BAX, Homo Mutant (A/A), Hetero (G/A), and Homo wild (G/G); and (c) HER2, Homo wild (A/A), Homo mutant (G/G), and Hetero (A/G).

After PCR reaction, 10 *μ*l of each PCR product was digested with different restriction enzymes as shown in Table 1 at 37⁰C for overnight. In the case of BCL-2 polymorphism (-938C>A), after digestion wild-type allele (CC) yielded two bands of 189 and 111 bp; wild-type/variant allele (CA) yielded 111, 189, and 300 bp and the variant allele (AA) yielded a single 300 bp band. For BAX polymorphism (-248G>A), after digestion wild-type allele (GG) yielded two bands (89 and 20 bp); wild-type/variant allele (GA) yielded 20, 89, and 109 bp, and the variant allele (AA) yielded a single 109 bp band. In the case of HER2 polymorphism (I655V, i.e., A>G), after digestion wild-type allele (AA) produced one band (148 bp); wild-type/variant allele (AG) produced 116, 32, and 148 bp, and the variant allele (GG) produced two bands 116 and 32 bp band. The digested PCR products were visualized on a 2% agarose gel containing ethidium bromide. PCR primers, PCR product sizes, restriction enzymes, and enzyme digests are listed in Table 1 and Figures 1(a)–1(c).

### 2.3. Statistical Analysis

Chi-square test was applied for comparing genotype and allele frequencies for statistical significance between breast cancer patients and controls. Observed and expected genotype frequencies of BCL-2, BAX, and HER2 gene polymorphism in controls showed no deviation from Hardy-Weinberg equilibrium. Chi-square test showed that there was no significant deviation from Hardy-Weinberg equilibrium for BCL-2, BAX, and HER2 SNP genotypes (*p* > 0.05). Odds ratios (ORs) with corresponding 95% confidence intervals (CIs) were determined to assess the strength of association of BCL-2 (-938C>A) and BAX (-248G>A) and HER2 Ile655Val polymorphism with breast cancer risk. Statistical significance was set at *p* < 0.05.

## 3. Results

### 3.1. Association of BCL-2 (-938C>A) Polymorphism with Breast Cancer

The genotype and allele frequencies of BCL-2 (-938C>A) polymorphism in cases and control are summarized in Table 2. The frequencies of CC, AC, and AA genotypes were 29.05%, 47.86%, and 23.07% in cases and 28.84%, 49.03%, and 22.11% in controls, respectively. The statistical analysis of observed genotypic frequencies did not show significant association (*p*-0.980). Similarly, there was no significant difference in allele frequencies between cases and control (*p*-0.937). Also, we did not find any significant association between BCL-2(-938C>A) polymorphism and breast cancer risk under recessive, dominant, and codominant models.

### 3.2. Association of BAX (-248G>A) Polymorphism with Breast Cancer

The frequencies of GG, AG, and AA genotypes in cases and controls were 79.48%, 17.09%, and 3.41%, and 77.88%, 18.26%, and 3.84%, respectively (Table 3). The statistical analysis of observed genotypic frequencies did not show significant association (*p*-0.956). Similarly, no significant difference was observed in allele frequencies between cases and control (*p*-0.747). Also, there was no significant relationship between BAX (-248G>A) polymorphism and risk of breast cancer under recessive, dominant and codominant models.

### 3.3. Association of HER2 Ile655Val Polymorphism with Breast Cancer

The genotype and allele frequencies of HER2 Ile655Val polymorphism in cases and control are summarized in Table 4. The genotype frequencies for Ile/Ile (A/A), Ile/Val (A/G), and Val/Val (G/G) were 55.55%, 37.60%, and 6.83% in cases and 75.96%, 21.15%, and 2.88% in controls, respectively. With reference to Ile/Ile (A/A) genotype, frequency of Ile/Val (A/G) genotype was significantly higher among cases than in control group and observed to increase the breast cancer risk (OR, 2.43; 95% CI, 1.32–4.46; p-0.004). The statistical analysis of observed genotypic frequencies showed significant association (p-0.0059). The frequency of Val (G) allele was significantly higher in cases as compared to controls (6.83% vs 2.88%, resp.). Compared to Ile (A) allele, significant increase in the risk of breast cancer was observed with Val (G) allele (OR, 2.21; 95% CI, 1.35–3.63; p-0.0016).We observed significant association between HER2 Ile655Val polymorphism and breast cancer risk under the dominant (OR = 2.52; 95% CI: 1.41–4.51; *p*-0.001) and codominant (OR, 2.24; 95% CI: 1.23–4.09; *p*-0.008) model, whereas no significant relationship was found under the recessive model (OR, 2.47; 95% CI: 0.63–9.57; *p*-0.190).

### 3.4. Relationship of BCL-2 (−938C>A), BAX (−248G>A), and HER2 Ile655Val Polymorphism with Tumor Grade

In this present study, we reported no significant association of the BCL-2 (−938C>A), BAX (−248G>A), and HER2 Ile655Val polymorphism with tumor grade (Table 5).

## 4. Discussion

Apoptosis is highly programmed cell death and has a significant role in functionality and development of multicellular organism. Damaged and redundant cells are eliminated by activation of apoptosis through various physiological or pathological death signals for maintaining homeostasis [14]. Apoptosis can be attained through two main pathways: mitochondrial pathway and death-receptor pathway and both are propagated through a caspase cascade which results into activation of apoptosis [15, 16]. During carcinogenesis, apoptosis is evaded by three different mechanisms: caspase activity loss, disturbed death receptors signaling, and imbalance between proapoptotic and antiapoptotic proteins [17–20].

BCL-2 protein plays significant function in the regulation of apoptosis and cell cycle delay. BCL-2 overexpression is found to be associated with different types of cancers such as prostate cancer, chronic lymphocytic leukemia, non-small cell lung cancer, breast cancer, esophageal cancer, lung cancer, and endometrial cancer [21–25]. Dysregulation of apoptosis due to imbalances in BAX/BCL-2 levels may result in breast cancer pathogenesis [26]. In our study, there was no significance difference in the allelic and genotype frequency of the BCL-2 (−938C>A) polymorphism between cases and controls. We observed no significant relationship between BCL-2 (−938C>A) polymorphism and risk of breast cancer under recessive, dominant, and codominant model. Our results showed that BCL-2 (−938C>A) polymorphism was not associated with breast cancer risk. The findings of our study showed discrepancy from a study from Hyderabad, India, which reported the association of AA genotype with increased risk (AAVs AC + CC) for breast cancer by 2.86-fold (p-0.07) and the frequency of A allele was also increased in the breast cancer cases than in controls (95 % CI, 1.41 (0.97–2.04) p-0.06) [14]. Similarly, another study also found that AA genotype of BCL-2 (−938C>A) may be associated with breast cancer susceptibility and increase the breast cancer risk in Chinese women [27], which was also inconsistent with our findings.

BAX is a proapoptotic protein which controls apoptosis through regulation of mitochondrial outer membrane permeabilization [28]. In numerous cancers, protein expression and function are found to be affected by mutations in the promoter and coding regions of the BAX gene [29]. Genetic alterations in the BAX gene may play important role in cancer initiation and progression as it contains series of target genes involving various tumor suppressor genes and oncogenes [30–34]. In the current study, we did not observe statistically significant difference in the genotype and allele frequencies of BAX (−248G>A) polymorphism among cases and control. No significant association was found between BAX (−248G>A) polymorphism and breast cancer under recessive, dominant, and codominant model. We failed to find an association between BAX (−248G>A) polymorphism and breast cancer risk. Our results were in concordance with a study conducted by Yildiz et al. [35] where no significant difference was observed in genotype and allele frequencies for BAX(−248G>A) among breast cancer patients and controls in Turkish women. Similarly, a meta-analysis study conducted by Sahu and Choudhuri on seven independent case-control studies (1772 cases and 1708 controls) did not find any association of BAX(−248G>A) genotype and allele frequency with human cancer risk under different genetic models [36].

SNP at codon 655 of the HER2 gene shows isoleucine (ATC) to valine (GTC) substitution (I655V) in the transmembrane domain-coding region and was found to be associated with breast cancer risk [37]. HER2 belongs to epidermal growth factor receptor (EGFR) family and has intrinsic tyrosine kinase activity [38]. The members of this family regulate various cellular functions like differentiation and proliferation as they play significant function in signal transduction pathway [39]. Dimerization of the HER receptors leads to the activation of signaling pathways [40]. HER2 appears to be the favored heterodimerization partner for all HER members [41]. HER2 triggers various cellular signaling pathways involving mitogen-activated protein kinase (MAPK) and phosphatidylinositol 3-kinase (PI3K) cascades [42]. In our study, the allelic frequency and genotype distribution of HER2 Ile655Val polymorphism exhibited significant difference between cases and controls. We found significant association between HER2 Ile655Val polymorphism and breast cancer risk under the dominant and codominant model. This present study is the first one to show significant association between HER2 Ile655Val polymorphism and risk of breast cancer in Indian population, suggesting the potential role of this polymorphism in development of breast cancer. Previously, a meta-analysis study by Tao et al. [43] in overall analysis found that Val allele frequency was significantly higher in breast cancer cases than in controls (OR = 1.1, 95% CI 1–1.2, p*-*0.04) on 20 eligible reports of 10,642 cases and 11,259 controls. Xie et al. [37] also reported that HER2 Ile655Val polymorphism may be a susceptibility biomarker for breast cancer among younger Chinese women. Furthermore, finding of our study was in accordance with previous studies in which presence of Val allele in HER2 polymorphism was associated with breast cancer risk among Portuguese [44] and Slovak populations [45].

In the Brazilian population, HER2 Ile655Val polymorphism was suggested as a candidate marker for breast cancer susceptibility, although negatively associated with breast cancer susceptibility [46]. Similarly, Parvin et al. [47] showed association of HER2 rs1136201 polymorphisms with breast cancer in Bangladesh population. Moreover, Ozturk et al. [48] also suggested Ile/Val genotype of HER2 may act as a genetic risk factor for breast cancer in Turkish population.

Our finding was inconsistent with the previous studies which did not find any association of Her2 Ile655Val gene polymorphisms with the breast cancer risk in Turkish [49–51], Korean [52], Malaysian [53], and Iranian [54] populations. Many studies suggested that HER2V655 allele is not a risk factor for breast cancer in British population [55] and Caucasians, African–Americans, or Latinas [56]. Another meta-analysis study by Dahabreh and Murray also reported no association between HER2 Ile655Val polymorphism and breast cancer development which was based on 33 case-control studies including 20,461 cases and 23,832 controls [57]. Likewise, in a previous study from our group [58], we found no significant association of HER2 Ile655Val polymorphism with colorectal cancer in Indian population.

There were some limitations in the present study. Firstly, the sample size was small. Indian population is thought to be most diverse due to different sociocultural traditions. A single larger study with diverse sample size may help us in better understanding the association of the genetic variation of these genes with breast cancer risk. Secondly, the gene-environment and gene-gene interactions have not been taken into account. Combination of gene-environment interactions and gene polymorphisms should be taken into consideration to better understand the genetic background of breast cancer. Further studies on larger sample size are needed to confirm our findings.

## 5. Conclusion

In conclusion, the present case-control study concludes that BCL-2 (-938C>A) and BAX (-248G>A) polymorphism were not significantly associated with breast cancer risk. This current study for the first time revealed significant association of HER2 Ile655Val polymorphism with high risk of breast cancer in Indian population. These genetic risk factors identification can be useful in predicting the occurrence of breast cancer and defining high risk individuals. Hence, we suggest that each population need to evaluate its own genetic profile for breast cancer risk that may be helpful for better understanding the racial and geographic differences reported for breast cancer incidence and mortality.

## Figures and Tables

**Table 1:** PCR primers, PCR product sizes, restriction enzymes, and enzyme digests of BCL-2 (-938C>A), BAX (-248G>A), and HER2 Ile655Val genes.

Gene	Position and base change	Genotyping	Primer	PCR product	Restriction enzyme used	Enzyme digests
BCL-2	-938C>A	PCR-RFLP	5′-CTGCCTTCATTTAT CCAGCA-3′ (forward) 5′-GGCGGCAGATGA ATTACAA-3′ (reverse)	300 bp	*BccI (1 unit)*	C allele: 189 and 111 bp; A allele: 300 bp

BAX	-248G>A	PCR-RFLP	5′-CATTAGAGCTGCGA TTGGACCG-3′ (forward) 5′-GCTCCCTCGGGAG GTTTGGT-3′ (reverse)	109 bp	*MspI (1 unit)*	G allele: 89 and 20 bp A allele: 109 bp

HER2	I655V A>G	PCR-RFLP	5′-AGAGCGCCAGCCCTCT GACGTCCAT-3′ (forward) 5′-TCCGTTTCCTGCAGCA GTCTCCGCA-3′ (reverse)	148 bp	*BsmAI (1 unit)*	G allele: 116 and 32 bp; A allele: 148 bp

**Table 2:** Genotype distribution, allele frequency, and association analysis of BCL-2(-938C>A) polymorphism and risk of breast cancer under different genetic models.

Genotype/allele		Cases (*n* = 117)	Control (*n* = 104)	Odd ratio (95% CI)	*p* value
CC		34 (29.05%)	30 (28.84%)	Ref	Ref
AC		56 (47.86%)	51 (49.03%)	0.968 (0.521–1.801)	0.920
AA		27(23.07%)	23 (22.11%)	1.035(0.493–2.175)	0.925
		*p* value 0.980	
Recessive model	AA	27	23	1.05 (0.56–1.98)	0.864
	AC+CC	90	81		
Dominant model	AC+AA	83	74	0.98 (0.55–1.77)	0.972
	CC	34	30		
Codominant model	AC	56	51	0.95 (0.56–1.61)	0.861
	CC+AA	61	53		
Allele					
C		124 (52.99%)	111 (53.37%)	1.01 (0.69–1.47)	0.937
A		110 (47.01%)	97 (46.63%)		

OR: odds ratio, CI: confidence interval, and *n*: number of samples.

**Table 3:** Genotype distribution, allele frequency, and association analysis of BAX(-248G>A) polymorphism and risk of breast cancer under different genetic models.

Genotype/allele		Cases (*n* = 117)	Control (*n* = 104)	Odd ratio (95% CI)	*p* value
GG		93 (79.48%)	81 (77.88%)	Ref	Ref
AG		20 (17.09%)	19 (18.26%)	0.916 (0.457–1.837)	0.806
AA		4 (3.41%)	4 (3.84%)	0.871 (0.211–3.594)	0.848
		*p* value -0.956	
Recessive model	AA	4	4	0.88 (0.21–3.63)	0.865
	AG + GG	113	100		
Dominant model	AG + AA	24	23	0.90 (0.47 to 1.73)	0.771
	GG	93	81		
Codominant model	AG	20	19	0.92 (0.46–1.84)	0.819
	GG + AA	97	85		
Allele					
G		206 (88.03%)	181 (87.02%)	0.91 (0.51 to 1.60)	0.747
A		28 (11.97%)	27 (12.98%)		

OR: odds ratio, CI: confidence interval, and *n*: number of samples.

**Table 4:** Genotype distribution, allele frequency, and association analysis of HER2 Ile655Val polymorphism and risk of breast cancer under different genetic models.

Genotype/allele		Cases (*n* = 117)	Control (*n* = 104)	Odd ratio (95% CI)	*p* value
Ile(A)/Ile(A)		65 (55.55%)	79 (75.96%)	Ref	Ref
Ile(A)/Val(G)		44 (37.60%)	22 (21.15%)	2.43 (1.32–4.46)	0.004^∗^
Val(G)/Val(G)		8 (6.83%)	3 (2.88%)	3.24 (0.82–12.7)	0.091
		*p* value 0.0059^*∗*^	
Recessive model	GG	8	3	2.47 (0.63–9.57)	0.190
	AG + AA	109	101		
Dominant model	AG + GG	52	25	2.52 (1.41–4.51)	0.001^*∗*^
	AA	65	79		
Codominant model	AG	44	22	2.24 (1.23–4.09)	0.008^*∗*^
	AA + GG	73	82		
Allele					
Ile (A)		174 (74.36%)	180 (86.54%)	2.21 (1.35–3.63)	0.0016^*∗*^
Val (G)		60 (25.64%)	28 (13.46%)		

*n*: number of samples, OR: odds ratio, and CI: confidence interval. ^*∗*^Significant at *p* < 0.05.

**Table 5:** Association of BCL-2 (-938C>A), BAX (-248G>A), and HER2 Ile655Val polymorphism with tumor grade.

	Genotype	Tumor grade I	Tumor grade II	Tumor grade III	*p* value
BCL-2 (-938C>A)	C/C	10 (30.30%)	17 (28.33%)	7 (29.16%)	0.980
	A carrier (AC + AA)	23 (69.69%)	43 (71.66%)	17 (70.83%)	

BAX (-248G>A)	G/G	24 (72.72%)	47 (78.33%)	22 (91.67%)	0.206
	A carrier (AG + AA)	9 (27.27%)	13 (21.66%)	2 (8.33%)	

HER2 Ile655Val	Ile(A)/Ile(A)	21 (63.64%)	32 (53.33%)	12 (50%)	0.523
	Val (G) carrier (AG + GG)	12 (36.36%)	28 (46.66%)	12 (50%)	

## Data Availability

The datasets used and/or analyzed during the present study are available from the corresponding author.

## Abbreviations

BCL-2: B -cell leukemia/lymphoma 2
BAX: BCL-2-associated X protein
HER2: Human epidermal growth factor receptor 2
IDC: Infiltrating ductal carcinoma
NOS: Not otherwise specified
PCR-RFLP: Polymerase chain reaction-restriction fragment length polymorphism
OR: Odds ratio
CI: Confidence interval
SNP: Single nucleotide polymorphism
EGFR: Epidermal growth factor receptor
MAPK: Mitogen-activated protein kinase
PI3K: Phosphatidylinositol 3-kinase.

## Additional Points

(i) BCL-2 (-938C>A) and BAX (-248G>A) polymorphism were not associated with breast cancer risk. (ii) Significant association of HER2 Ile655Val polymorphism with risk of breast cancer indicates the potential role of this polymorphism in development of breast cancer. (iii) No significant association of the BCL-2 (-938C>A), BAX (-248G>A), and HER2 Ile655Val polymorphism was found with tumor grade.
